# Clinical report of six-month follow-up after cementing PEEK crown on molars

**DOI:** 10.1038/s41598-022-23458-5

**Published:** 2022-11-09

**Authors:** Hitomi Kimura, Koji Morita, Fumiko Nishio, Hitoshi Abekura, Kazuhiro Tsuga

**Affiliations:** 1 Department of Advanced Prosthodontics, Hiroshima University Graduate School of Biomedical and Health Sciences, Hiroshima, Japan; 2 Department of Fixed Prosthodontics, Kagoshima University Graduate School of Medical and Dental Sciences, Kagoshima, Japan

**Keywords:** Health care, Medical research

## Abstract

We conducted a six-month clinical follow-up on computer-aided design/computer-aided manufacturing-fabricated molar polyetheretherketone PEEK crowns to investigate their therapeutic effect. Only the PEEK crowns were examined as our study focused on short-term clinical evaluation of the new PEEK material. Twenty-three cases of PEEK crowns placed on the molars of 20 subjects (7 males and 13 females, mean age: 60.6 ± 14.2 years) were included in the study. The evaluation items were the condition of the crowns at the time of cementation and after six months, patient satisfaction, masticatory ability, and occlusal force. Mann–Whitney *U* tests with a significance level of 5% were used to examine the difference in glucose concentration by masticatory ability, occlusal pressure, and occlusal force, with and without PEEK crowns. The occlusion, margin fit, and contact of all 23 cases at the time of cementing were good. Six months after cementation, there was no crown desorption, fracture or crack, and prosthodontics was not needed in the 22 cases (one patient dropped out). No wear of the dental antagonist was observed. Patient satisfaction was generally high. There was no significant difference in masticatory ability between the groups with and without PEEK crowns. The subject's occlusal force was within the normal range. PEEK crowns used on molars can replace metal crowns and hold promise for an appropriate and effective treatment.

## Introduction

In the past few years, the use of metals in crown prostheses has decreased because of the aesthetics, metal allergies and soaring prices of precious metals^1–4^. In addition, since precious metals are getting expensive, there is an urgent need to introduce materials with fewer price fluctuations^5^. Hybrid resin and zirconia are often used as non-metal crown prosthetic materials, but the hybrid resin is prone to fracture and delamination^6,7^, and zirconia is prone to damage by the antagonistic portion of tooth owing to its extreme hardness^8,9^. Therefore, we focused on polyetheretherketone (PEEK), a high-performance and thermostability for plastic material with excellent mechanical strength, wear and water absorption resistance, chemical resistance, biosafety, and processability as an adaptable dental material. As a thermoplastic resin, PEEK is used in automobile and aircraft parts that require durability. It has been applied to spinal implants in clinical medicine, and its biological safety has been confirmed^10,11^.

However, since PEEK has a grayish beige color tone and low bond strength with cement, it is not frequently used as a crown material^12^. To solve the above problems, the color of PEEK is changed to milky white by adding 20wt% titanium dioxide, which reduces the risk of metal allergy compared to other metals^13^. Moreover, the bond strength is increased by various surface treatments such as high-concentration sulfuric acid treatment, sandblasting, or laser grooving^14–17^. A few studies have achieved clinically applicable bond strengths by using appropriate surface treatments, primers, and types of cement^18–23^. Therefore, we applied to the Hiroshima University Clinical Research Ethics Review Committee for a clinical study of PEEK crowns for molar teeth fabricated with a computer-aided design/computer-aided manufacturing (CAD)/CAM system (Clinical assessment of PEEK crown for molar teeth: jRCTs062180040), after which a clinical study was conducted at the Hiroshima University Hospital.

PEEK is one of the promising non-metallic materials for crown restorations, but studies on its clinical applications are rare. Therefore, in this exploratory study, we reported the clinical evaluation of 22 PEEK crowns for molar teeth at a six-month follow-up after cementation.

## Materials and methods

### Subjects

This study was approved by the Hiroshima University Clinical Research Ethics Review Committee (Clinical assessment of PEEK crown for molar teeth: jRCTs062180040) and in compliance with the Good Clinical Practice Guideline of the International Conference on Harmonization (ICH-GCP)^24^. This clinical study was exploratory research of a prospective cohort with a six-month follow-up conducted at the Hiroshima University Hospital Dentistry from March 29, 2018, to October 2020. The subjects of this study were 23 cases of PEEK crowns for molar teeth in 20 patients (7 males and 13 females; age range, 23–82 years; mean age, 60.6 ± 14.2 years) who required prosthetic treatment with a single crown in the molars after treatment for dental caries or apical periodontitis. All patients provided written and verbal informed consent for prosthetic treatment with PEEK crowns.

### Inclusion and exclusion criteria

Inclusion criteria were as follows: (i). Age: > 20 years, (ii). Dental antagonists to test teeth for the single crown of the upper and lower molars (possibility of two consecutive single crowns). Abutment teeth height of approximately 3 mm on the buccal and lingual sides after preparation. The exclusion criteria were as follows: (i) abutment tooth for removable partial dentures, (ii) severe periodontal disease (the criteria included pocket of ≥ 6 mm, bone resorption/attachment loss of root length ≥ 1/2, and root bifurcation lesion of level ≥ 2). Patients who met these criteria (i) or (ii), were excluded from the study population.

### Materials

The PEEK material used in this study was Vestakeep DC4450 PEEK (tooth-colored/polyetheretherketone, 20wt% titanium dioxide pigments) (Daical-Evonik Ltd., Tokyo). The biocompatibility of PEEK materials is good^11^. The PEEK material used here meets the criteria for in vivo use (https://medical.evonik.com/en/high-performance-polymers/vestakeep-peek/biocompatibility). In other field, PEEK can be implanted in subcutaneous or submucosal tissues in the jawbone and spine of the human body, and its biosafety as an intraosseous implant in contact with cellular tissue is guaranteed. In the present study, the PEEK crowns were applied in vivo in the oral cavity, but these were not implanted in submucosal tissue. Since these were used internally but outside of the body cavity, were not in direct contact with living cell or tissue and are not considered biohazardous.

Visio. Link (Bredent, Chesterfield) was selected based on the report that sandblasting the PEEK surface and Visio link, a primer, improves adhesive cement bonding and is used for the inner side of the PEEK crown^19^.

The following primers were used for the surfaces of abutment teeth: V-primer (Sun Medical, Moriyama) for metal core^25^, Super-bond PZ primer (Sun Medical, Moriyama) for resin core^26^, and Scotchbond Universal Adhesive (3 M ESPE, Neuss) for metal and resin cores^27^.

Two types of adhesive resin cement, MMA-based Super-Bond C&B (Sun Medical, Moriyama) and composite-based RelyX Ultimate Resin Cement (3 M ESPE, Neuss), were used for final cementing and verifying whether this would result in detachment or breakage.

### Surgical protocol

After taking preliminary impressions and doing preoperative examinations to check the antagonist teeth, metalcore or resin core abutments were fabricated as per the requirement. Thereafter, the abutment teeth were formed according to a method reported previously^28^. The basic abutment tooth geometry was like that of a full cast crown, with occlusal surface clearance to the antagonistic tooth of at least 1.0 to 1.5 mm. The buccal, adjacent, and lingual axial surfaces clearance were at least 1.0 mm at the center and about 0.8 mm near the margin. The corner of the transition between the axial and occlusal surfaces was rounded, and the finish line was smooth. Relative axial surfaces near the cervical side were within 20 degrees. Abutment tooth formation was performed using a turbine head with a diamond bar and the impression was taken using a custom tray and a silicone impression material. Dentists with > 5 years of experience in prosthetic treatment were registered as per the research protocol for performing the procedure. Hard plaster was then injected into the impression and a working model was made. The antagonistic dentition model was impressed with alginate impression material and the maxillomandibular relationship of intercuspal position was recorded using a silicon bite.

**Figure 1:**
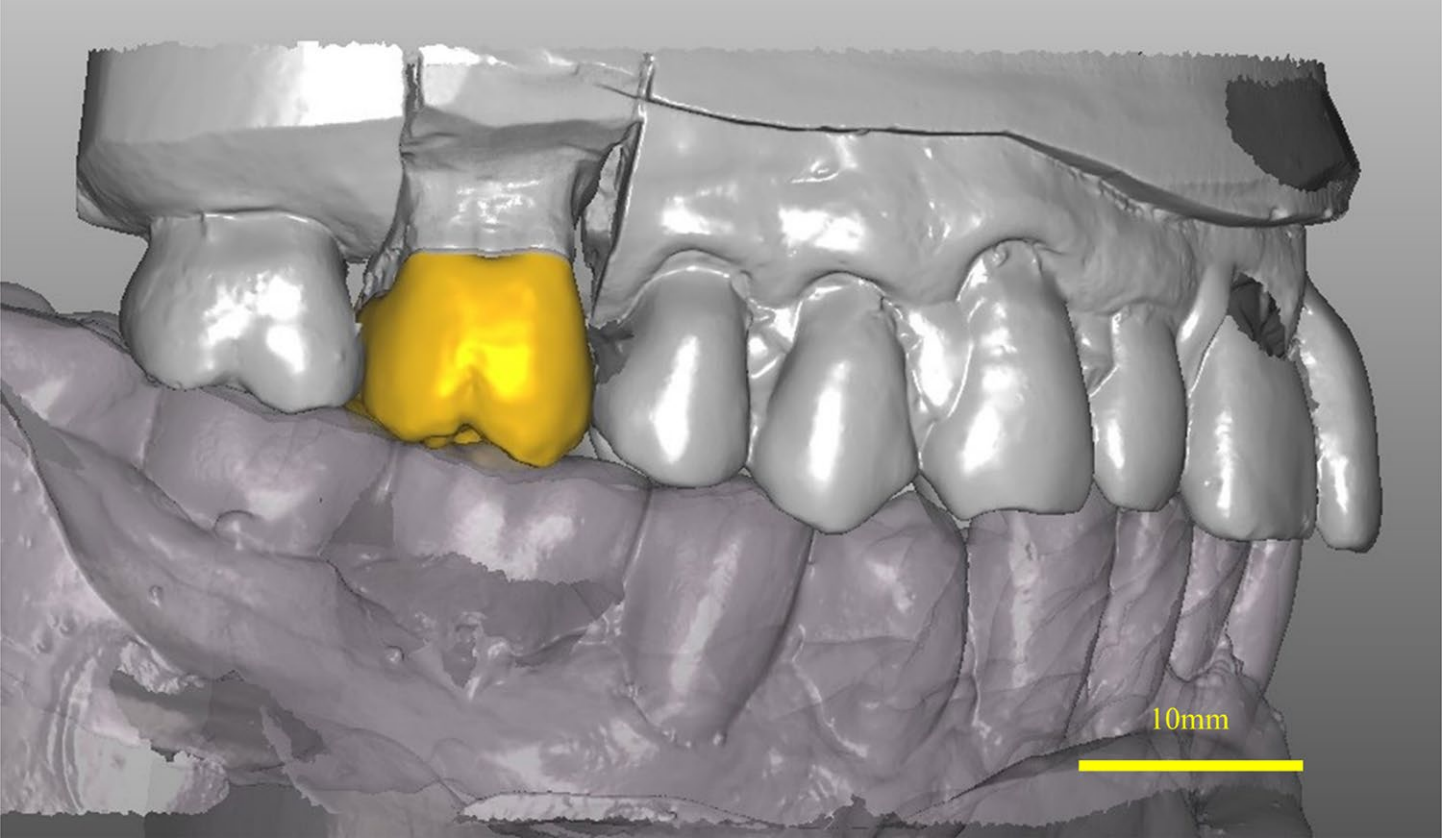
Computer-aided design of polyetheretherketone crown. *CAD design of a PEEK crown on an upper right first molar to harmonize the occlusal contact with the antagonists and the contact relationship with the adjacent teeth.

**Figure 2:**
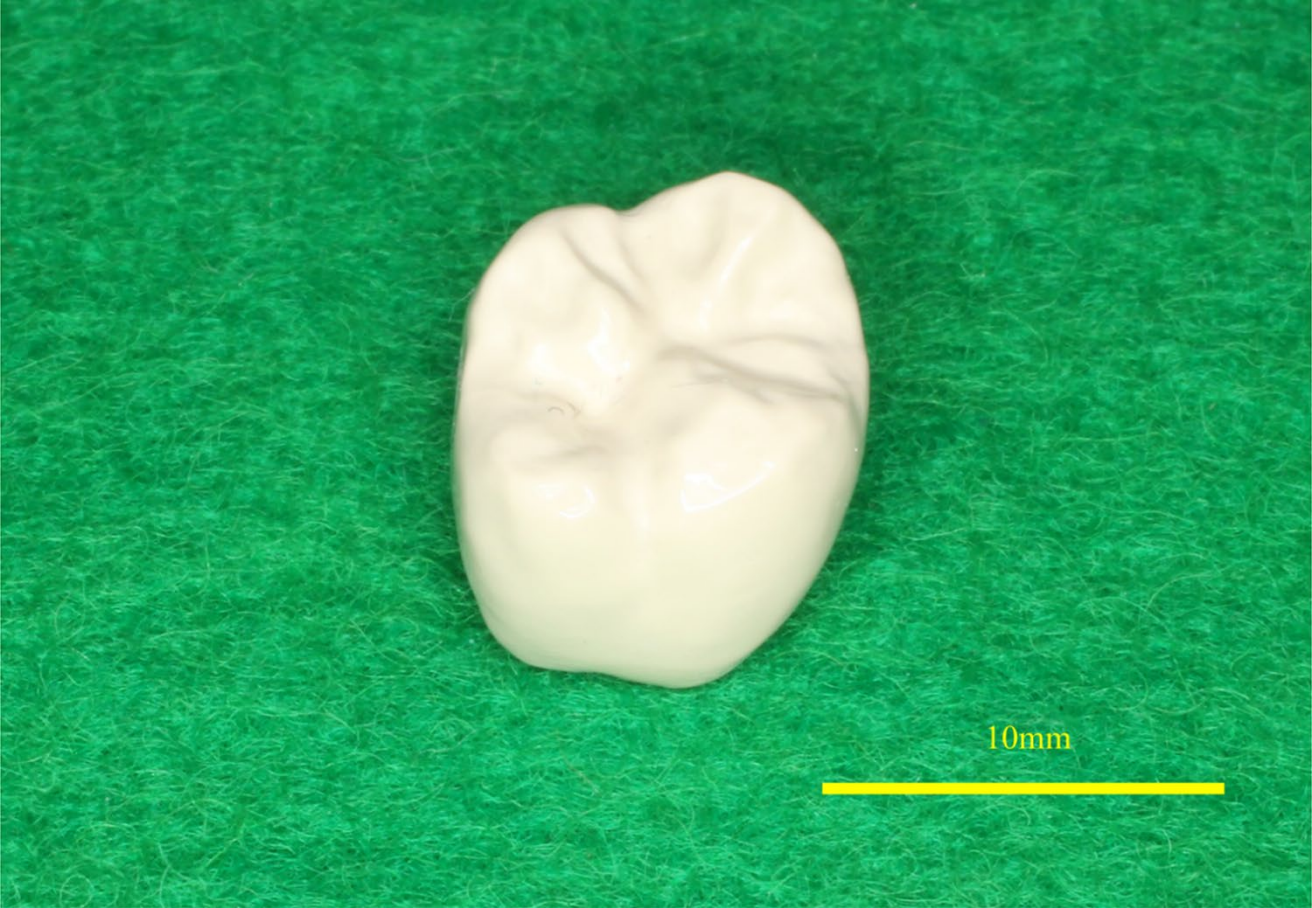
View of the occlusal surface of the completed polyetheretherketone crown. The crown designed by CAD in Fig. 1 was cut from a PEEK block using a milling machine and polished to a high gloss finish.

The abutment tooth model and working dental model were scanned and converted to 3D data. A computer-aided design/computer-aided manufacturing (CAD/CAM) system was used to fabricate PEEK crowns (Figs. 1 and 2). The CAD/CAM system consisted of a scanner (S600 ARTI SCANNER, Zirkonzahn Products, South Tyrol), milling machine (DWX-52D, SHOFU INC., Kyoto), and CAM software (WORKNC DENTAL https://open-dental.jp/lineup/worknc-dental/, Vero Software KK, Tokyo). After trial fitting, the adjacent and occlusal surfaces were adjusted and polished. Rough polishing of PEEK crowns was done with a carborundum point fine (Shofu INC, Kyoto), medium polishing with a big silicon point or silicon point M3 (Shofu INC, Kyoto), and final polishing with Tiger Poli (Bis Chemical Laboratory Co., Ltd. Osaka) using a buff. After polishing the PEEK crown, ultrasonic cleaning and ethyl alcohol cleaning were performed for disinfection.

The inner surface of the PEEK crown was sandblasted with alumina particles (Danville DA-15301 Aluminum Oxide, 50 Micron, Shape, White, Danville Engineering., San Ramon) at a spray distance of 10 mm, air pressure of 0.1 MPa, and spray time of 10 s. Later, Visio. link was applied, dried with air, and irradiated with light for 90 s. The two types of adhesive resin cement, MMA-based Super-Bond C&B and composite-based RelyX Ultimate Resin Cement, which are known to have promising adhesive strength with PEEK^16,18^, were used in a total of 23 cases (Super-Bond C&B: 11 cases, RelyX Ultimate Resin Cement: 12 cases). The PEEK material used in our study was similar to the one used in a previous study^18^. The PEEK surfaces were processed with sandblasting treatment and primer treatment with Visio. Link. Later, the two types of cement including RelyX Ultimate Resin Cement and Super-Bond C&B, were used, and shear bond strength was shown to be 10 MPa or higher. Based on this result, the treatment on the inner surface of PEEK crowns in this study was sandblasted and treated with Visio. Link primer.

After preparing the inner surface of the PEEK crown, the surfaces of the abutment teeth were treated with the recommended primer for cementation. In the case of Super-Bond C&B, V-primer was applied to the metal core, and Super-bond PZ primer to the resin core and dried. In the case of RelyX Ultimate Resin Cement, Scotchbond Universal Adhesive was applied to the metal and resin cores, dried, and then exposed to light for 20 s for bonding.

### Evaluation items and methods

The 23 cases of PEEK crowns were evaluated at the time of cementation and the 22 cases after six months of placement. The evaluation items included the following: the abutment morphology; the height of the abutment teeth and clearance to the occlusal tooth surface of antagonist before setting crown (for the space of occlusal thickness of PEEK crown); the condition of PEEK crown in the oral cavity at the time of cementation; occlusal contact, margin conformation, and adjacent surface contact condition; occlusal contact condition and the condition of PEEK crown in the oral cavity after six months; cracking, fracture, dehiscence, occlusal contact, pain, secondary caries, occlusal surface condition, surface texture, discoloration and staining, plaque adhesion, marginal gingiva, occlusal surface condition of the dental antagonist indicating changes in the condition of the surface of dental antagonist due to wear or occlusal wear, adjacent surface contact condition and crown condition for the need for retreatment, and patient satisfaction. In addition, masticatory ability^29–32^, occlusal force, and occlusal pressure were measured^33^ as oral functions.

The abutment morphology: To represent the abutment tooth height, the height of the occlusal plane was measured from the buccal, lingual, mesial, and distal sides of finish lines along with the tooth axis of the working model using digital calipers (Mitutoyo, Kawasaki). The clearance between the abutment and the opposing tooth was measured from the functional occlusal of the abutment to the central fossa of the opposing tooth of the working model using a clearance gauge (CeraSmart Clearance Gauge, GC, Tokyo).

The conditions of PEEK crown in the oral cavity: The occlusal contact, margin conformation, and adjacent surface contact condition at the time of cementation, cracking, fractures, dehiscence, secondary caries, occlusal surface condition, surface texture, discoloration and staining, marginal gingiva, occlusal surface condition of the dental antagonist, and the crown condition requiring retreatment after six months of cementation was determined by a dentist on visual examination. A single person performed this evaluation to avoid variation by several different raters. The criteria were established (Table 4) and slides were taken to ensure stability and avoid variation in evaluations at different times. Patient satisfaction was assessed by asking patients about improvement in chewing (chief complaint), aesthetic, and pain. The occlusal contact condition at the time of cementation was examined by one dentist using an articulating paper. The ideal occlusal condition was considered as "excellent”, the one that needed slight adjustment but was generally good as "good”, and the one that required extensive adjustment or was too large as "poor.” The occlusal contact status at six months after occlusal adjustment was assessed by one dentist using an articulating paper. The point contact at the functional occiput was considered as "no problem”, surface contact as "good”, and no contact as "loss”. The plaque adhesion was evaluated by one dentist using a mouth mirror and a probe. After drying the surface on the PEEK crown with compressed air, " Equivalent” was classified as plaque visually observed under artificial lighting^34^. The contact between the adjacent teeth and the crown was determined using a contact gauge (GC, Tokyo, Japan). Adjacent surface contact during cement bonding was determined using a 50 µm contact gauge. Intraoral photographs and X-rays were taken, if necessary, to check the condition of the PEEK crowns and abutment teeth.

### Evaluation of oral function

After cementing, the oral function was quantified by occlusal force and masticatory ability test in a month or three months. Occlusal force testing was performed using a film for measuring occlusal force (Dental Prescale II, GC) and bite force analysis software (Bite Force Analyzer Ver2.1.1 https://www.gcdental.co.jp/sys/data/item/1559/, GC, Tokyo) to measure occlusal pressure and occlusal force. For the measurement, a pressure-sensitive film was prepared on the oral cavity while the occlusal surface of the subject was kept horizontal, and the subject was asked to bite down with the maximum bite force for 3 s. Then the occlusal pressure and occlusal force were calculated by analyzing the change in color of the film by applying pressure and using analysis software^33^. To test masticatory ability, gummy jelly with standardized ingredients, shape, and glucosensors was used. Gummy jellies were glucose-added by the company by the company for the chewing ability measurement test and were offered as a standard product at 2.5 g each. Immediately after the subject chewed gummy jelly for 20 s, 10 ml of water was placed in the mouth, and all the liquid in the mouth, including gummy jelly, was spat out on the filtration mesh. The masticatory ability was calculated by measuring the glucose concentration from the spit-out liquid^29–32^. Measurements were performed three times: once for right-side chewing, once for left-side chewing, and once for free chewing. Since previous papers have reported that masticatory ability tests performed twice have shown no difference in the results of each test, the present study was also conducted with one measurement for each site^35^. The standard value was set at 150 mg/dL or higher^30^, which is within the healthy range for edentulous individuals.

### Statistical analysis

The statistical program used was BellCurve for Excel (Social Survey Research Information Co., Ltd.). Mann–Whitney *U* test (significance level of 5%) was used to examine the difference in glucose concentration by masticatory ability, occlusal pressure, and occlusal force between the wearing and non-wearing PEEK crowns sides in 20 subjects. Kruskal–Wallis tests was performed to evaluate differences in masticatory capacity, occlusal pressure, and occlusal force by cement type.

### Ethical approval

All procedures performed in this study involving human participants were in accordance with the 1964 Helsinki declaration and its later amendments or comparable ethical standards. This study was approved by Hiroshima University Clinical Research Ethics Review Committee (jRCTs062180040, Clinical evaluation of PEEK crowns for molars).

### Consent to participate

Informed consent was obtained from all participants included in this study.

## Results

### Subjects

A total of 20 subjects and 22 PEEK crown cases were included in the study (Table 1). Among these, one subject was lost to follow-up at six months, and the total number of cases cemented were 22 after six months.

**Table 1:** Details of data for 20 subjects and 23 PEEK crown cases.

No	Sex	Age	FDI	Dental antagonist
**SB**				
1	Male	67	16	Full metal crown
2	Female	51	26	Full metal crown
3	Female	70	17	Full metal crown
4	Female	47	46	Full metal crown
5	Female	41	47	Temporary crown
6	Female	78	17	Ceramic
7	Male	79	47	Hard resin teeth (Removable denture)
8	Male	79	48	Full metal crown
9	Female	67	16	Ceramic (Implant)
10	Male	82	16	Full metal crown
11	Male	23	47	Normal tooth
**RU**				
12	Female	41	17	PEEK crown
13	Male	64	16	Zirconia crown
14	Female	53	46	Full metal crown
15	Female	46	16	Metal inlay
16	Male	65	26	Zirconia crown
17	Male	58	37	Normal tooth
18	Female	67	26	Metal ceramic crown
19	Female	67	27	Metal ceramic crown
20	Female	63	27	Metal inlay
21	Female	50	47	Normal tooth
22	Female	76	16	Normal tooth
23	Female	64	16	Metal inlay

*SB* super-bond C&B, *RU* relyx ultimate resin cement.

### Surgical protocol

Eleven cases (case numbers: 1–11) had the antagonistic teeth of six full metal crowns, one temporary crown, two ceramic crowns, one hard resin tooth, and one natural tooth. The mean age of these 11 cases with Super-Bond C&B cement was 60.5 years, and the target teeth were four maxillary first molars, two maxillary second molars, one mandibular first molar, three mandibular second molars, and one mandibular third molar. Meanwhile, the mean age of the subjects in the 12 cases (case numbers:12–23) using RelyX Ultimate Resin Cement was 59.1 years, and the target teeth were six maxillary first molars, three maxillary second molars, one mandibular first molar, and two mandibular second molars. In addition, the opposite teeth for the target teeth were one full metal crowns, two metal ceramic crowns, three natural teeth, one PEEK crown, two zirconia crowns, and three metal inlays (Table 1).

### Evaluation items and methods

**Figure 3:**
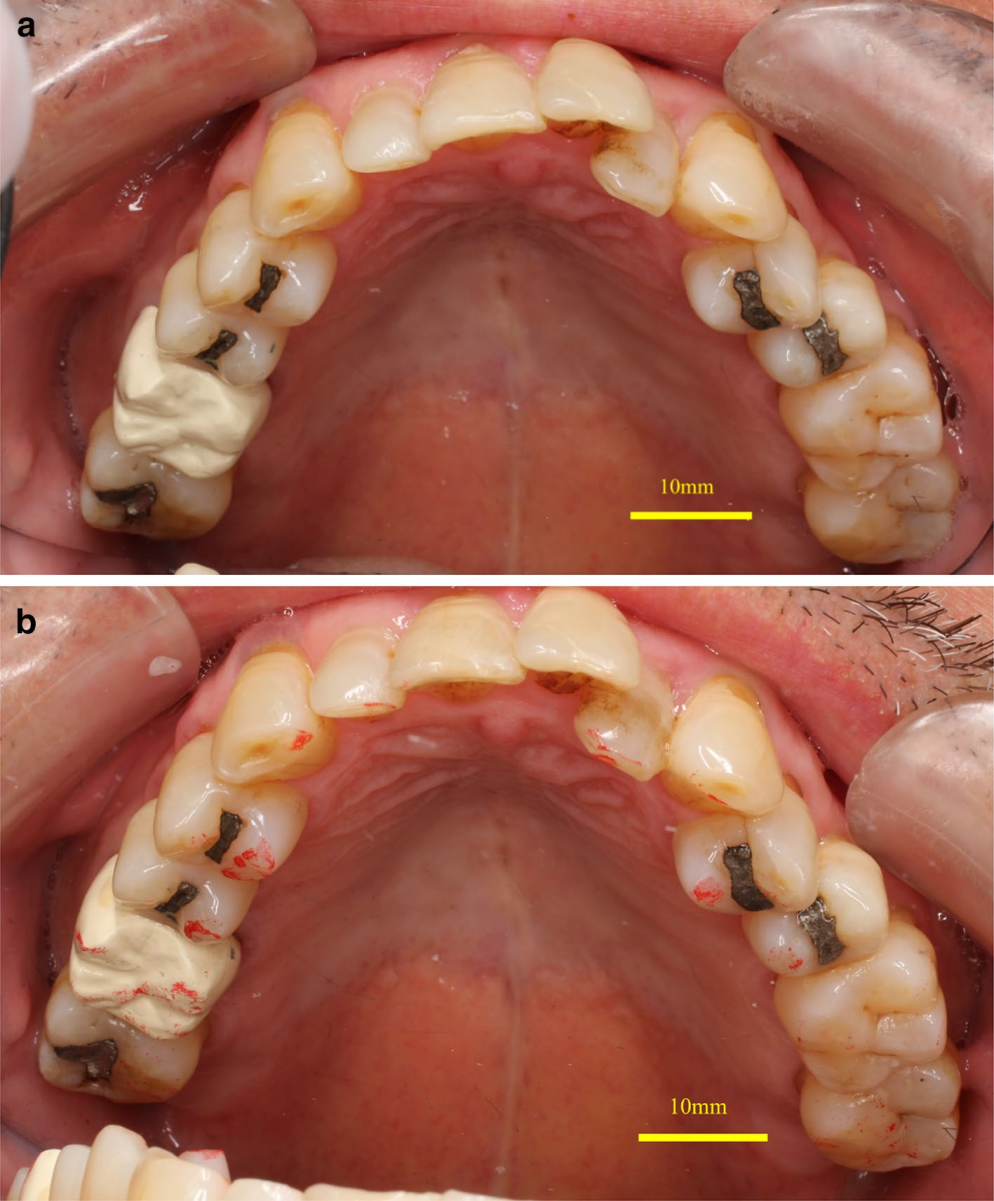
(**a**) Photographic image of an intraorally cemented polyetheretherketone crown on an upper right first molar at the time of cementing. (**b**) Photographic image of an intraorally cemented polyetheretherketone crown on an upper right first molar after six months of cementation.

**Figure 4:**
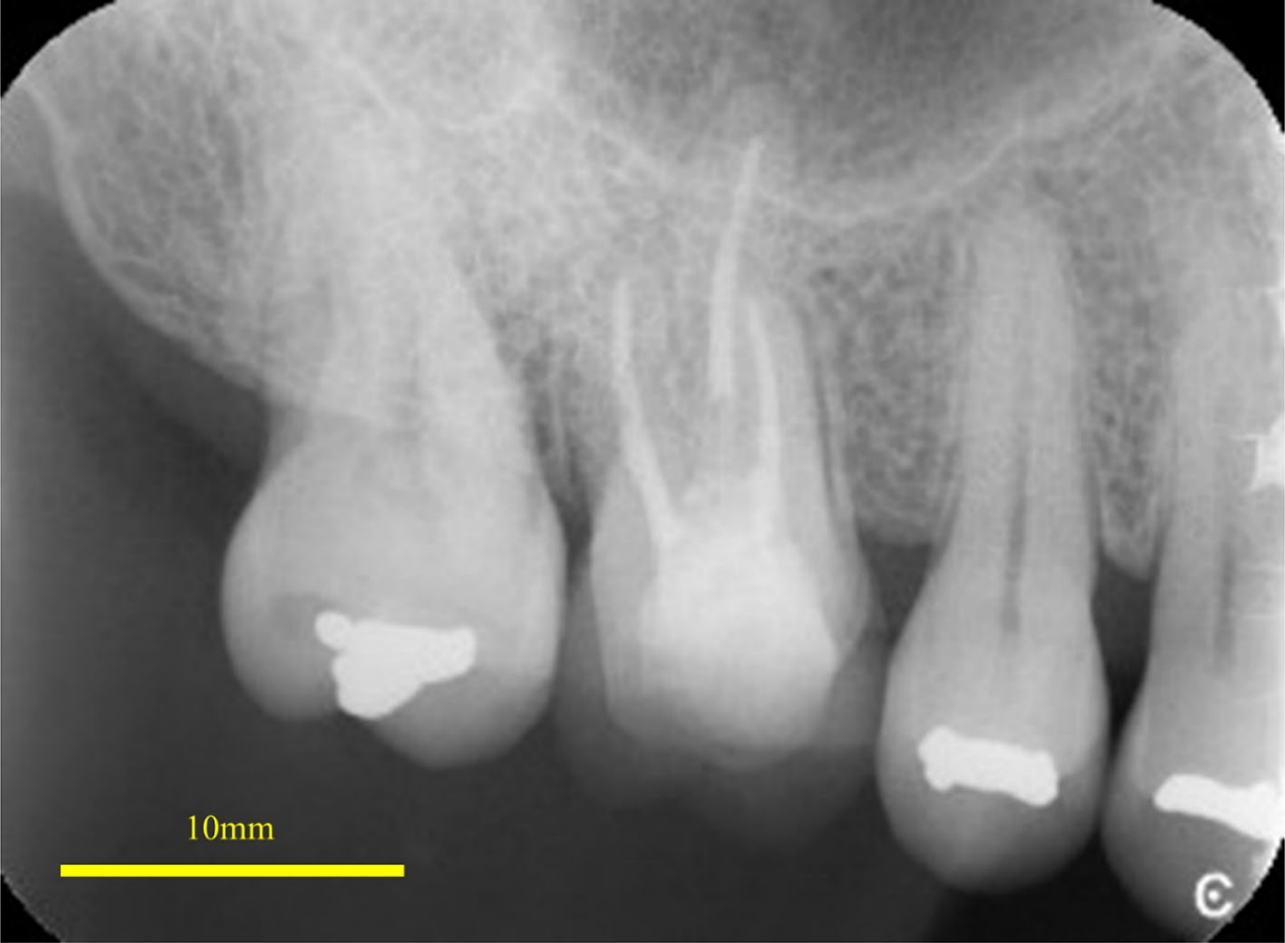
Digital X-ray image of PEEK crowns placed intraorally PEEK crown on the non-vital tooth with resin abutment and can be seen in the thin opaque image.

The abutment morphology: The measured height of the abutment teeth ranged from approximately 1.2 mm at the lowest site to approximately 7 mm at the highest site, and the clearance was 1.0 to 2.0 mm (Table 2). The color tone was milky white, as shown in one example of a PEEK crown on a maxillary right second molar (Fig. 3). The radiographs taken six months after placement showed that the PEEK crown was slightly opaque, and the outline of the crown was visible (Fig. 4). Among 23 PEEK crowns in 20 patients, twenty-two showed excellent contact. The margins of 21 cases were also in excellent condition, and no problems were found in the remaining cases (Table 3).

**Table 2:** Abutment morphology.

No	Abutment tooth height (mm)				Clearance (mm)
	B: Buccal side	L: Lingual side	M: Mesial side	D: Distal side	
**SB**					
1	B: 7.47	L: 5.15	M: 3.00	D: 5.80	2.0≦
2	B: 3.34	L: 4.19	M: 2.19	D: 2.47	1.5–2.0
3	B: 6.36	L: 4.61	M: 4.25	D: 3.76	1.5–2.0
4	B: 3.67	L: 3.12	M: 1.23	D: 1.48	1.5–2.0
5	B: 4.27	L: 2.99	M: 2.32	D: 2.65	1.5–2.0
6	B: 3.85	L: 3.93	M: 4.16	D: 3.17	1.0–1.5
7	B: 5.59	L: 4.16	M: 3.86	D: 5.44	2.0≦
8	B: 4.36	L: 3.86	M: 5.12	D: 2.36	1.5–2.0
9	B: 4.37	L: 4.75	M: 2.67	D: 2.30	2.0≦
10	B: 6.19	L: 4.78	M: 4.73	D: 5.01	1.5–2.0
11	B: 5.01	L: 3.06	M: 2.02	D: 4.40	1.5–2.0
**RU**					
12	B: 5.91	L: 3.90	M: 3.31	D: 2.72	1.0–1.5
13	B: 6.13	L: 4.02	M: 3.25	D: 4.82	2.0≦
14	B: 2.55	L: 3.11	M: 1.86	D: 2.22	1.0–1.5
15	B: 6.27	L: 5.07	M: 2.80	D: 2.93	2.0≦
16	B: 7.18	L: 5.39	M: 6.40	D: 4.86	2.0≦
17	B: 3.62	L: 2.37	M: 2.11	D: 1.22	2.0≦
18	B: 5.41	L: 5.92	M: 3.14	D: 3.86	2.0≦
19	B: 5.72	L: 3.47	M: 3.11	D: 3.66	2.0≦
20	B: 3.30	L: 4.17	M: 3.26	D: 1.64	2.0≦
21	B: 3.83	L: 2.92	M: 2.44	D: 1.40	1.0–1.5
22	B: 5.12	L: 3.55	M: 2.63	D: 2.69	1.0–1.5
23	B: 6.20	L: 3.53	M: 1.60	D: 3.28	1.0–1.5

*SB* super-bond C&B, *RU* relyX ultimate resin cement.

**Table 3:** Conditions of PEEK crown in the oral cavity at the time of cementing.

Evaluation items	Score		
	Excellent	Good	Poor
Occlusal contact*	22	1	0
Margin conformation**	21	2	0
Adjacent surface contact condition***	22	1	0

*Excellent: ideal occlusal condition, Good: needs slight adjustment but generally good, Poor: needs extensive adjustment or too large.

**Excellent: almost no pore space, Good: pore space is palpable, but there is little clinical problem, Poor: pore space is too large.

***Excellent: ideal contact condition, Good: contact position, form and strength are generally good, Poor: no contact or apparently inappropriate contact.

The conditions of PEEK crown in the oral cavity: The status of the PEEK crowns, abutment teeth, and dental antagonist after six months of placement is shown in Table 4. One of the subjects could not be followed up after six months of follow-up, but the remaining 19 subjects (22 cases) showed no detached fractures or cracks after six months of placement, and no cases required retreatment. In this study, two types of adhesive resin cement, composite resin and MMA cements, were used to investigate the occurrence of dehiscence and fracture after six months of wear. Debridement and severe breakage did not occur in all cases of our study. Therefore, no analysis was performed for different cements. There were light bite marks in two cases, slight surface roughness in six cases, slight staining in seven cases, and mild gingivitis in five cases (Table 5). The results of the satisfaction questionnaire are shown in Table 6. There were four comments each about chewing gum adhesion, but subjects’ satisfaction was generally high regarding recovery of the main complaint, mastication, and aesthetics.

**Table 4:** Conditions of PEEK crown in the oral cavity at six months after cementation.

Evaluation items	Score					
	A	B	C			
Cracking	22	0	–	A: None	B: Occurred	
Fracture	22	0	–	A: None	B: Occurred	
Dehiscence	22	0	–	A: None	B: Occurred	
Occlusal contact*	20	2	0	A: No problem	B: Good	C: Loss
Pain	22	0	–	A: No pain	B: Pain	
Secondary caries	22	0	–	A: None	B: Occurred	
Occlusal surface condition	20	2	0	A: No problem	B: Small attrition	C: Large attrition
Surface texture	16	6	0	A: No problem	B: Slight roughening	C: Crater formation
Discoloration/Stain	15	7	0	A: None	B: A little	C: Conspicuous
Plaque adhesion**	22	0	–	A: No plaque	B: Conspicuous	
Marginal gingiva	17	5	–	A: None	B: Mild inflammation	
Occlusal surface condition of the dental antagonist	22	0	0	A: No problem	B: Small attrition	C: Large attrition
Adjacent surface contact condition	22	0	–	A: None	B: Food impaction	
Crown condition for the need for retreatment	22	0	–	A: None	B: Occurred	

Assessment was done by one dentist using a mouth mirror and a probe. The surface of PEEK crowns were dried with compressed air and the plaque was visually examined under artificial light.

*No problem: point contact at the functional occiput, Good: surface contact at the functional occiput, Loss: no contact at the functional occiput.

**No plaque: No obvious plaque adhesion, Conspicuous: Obvious plaque adhesion.

**Table 5:** Details of score B evaluation items for six months after cementation.

Evaluation items	Score	Cement Type		Abutment tooth					antagonistic tooth								
	B	RelyX Ultimate Resin Cement	Super–Bond C&B cement	maxillary first molar	maxillary second molar	mandibular first molar	mandibular second molar	mandibular third molar	A	B	C	D	E	F	G	H	I
Occlusal contact	2	0	2	1	1	–	–	–	2								
Occlusal surface condition	2	1	1	2	–	–	–	–	1						1		
Surface texture	6	4	2	4	–	–	2	–	2	1			2		1		
Discoloration/Stain	7	6	1	5	–	–	2	–	1				2			2	2
Marginal gingiva	5	2	3	2	1	–	1	1	1		1					1	2

*A* full metal crown, *B* temporary crown, *C* ceramic crown, *D* hard resin teeth, *E* natural tooth, *F* PEEK crown, *G* zirconia crown, *H* partially covered metal crown, *I* ceramic-fired cast crown.

**Table 6:** Patient satisfaction at six months after cementation.

Evaluation items	Score		
	Excellent	Good	Poor
Improvement of chief complaint	21	1	0
Chewing	21	1	0
Aesthetic	13	9	0
Other	"Gum sticks" "I am worried about stain" (Male 1, Female:3)		

### Evaluation of oral function

**Figure 5:**
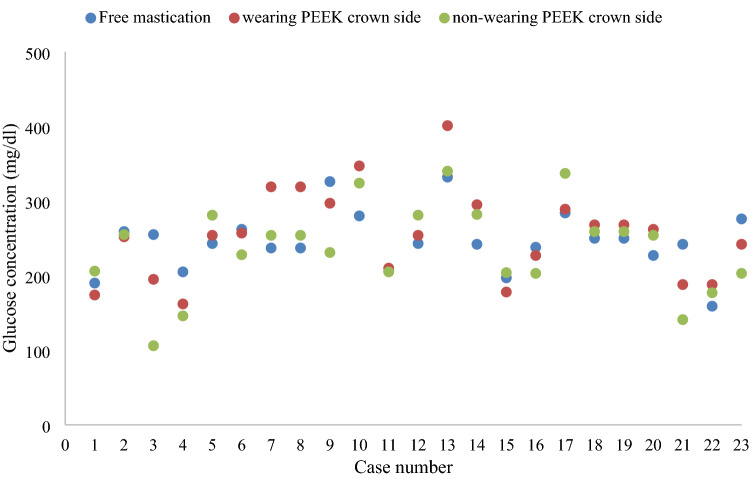
Distribution for the 23 cases of Glucose concentration by masticatory ability test focused on the mounting side of the PEEK crown. *Classified by wearing PEEK crowns side and non-wearing PEEK crowns sides.

**Figure 6:**
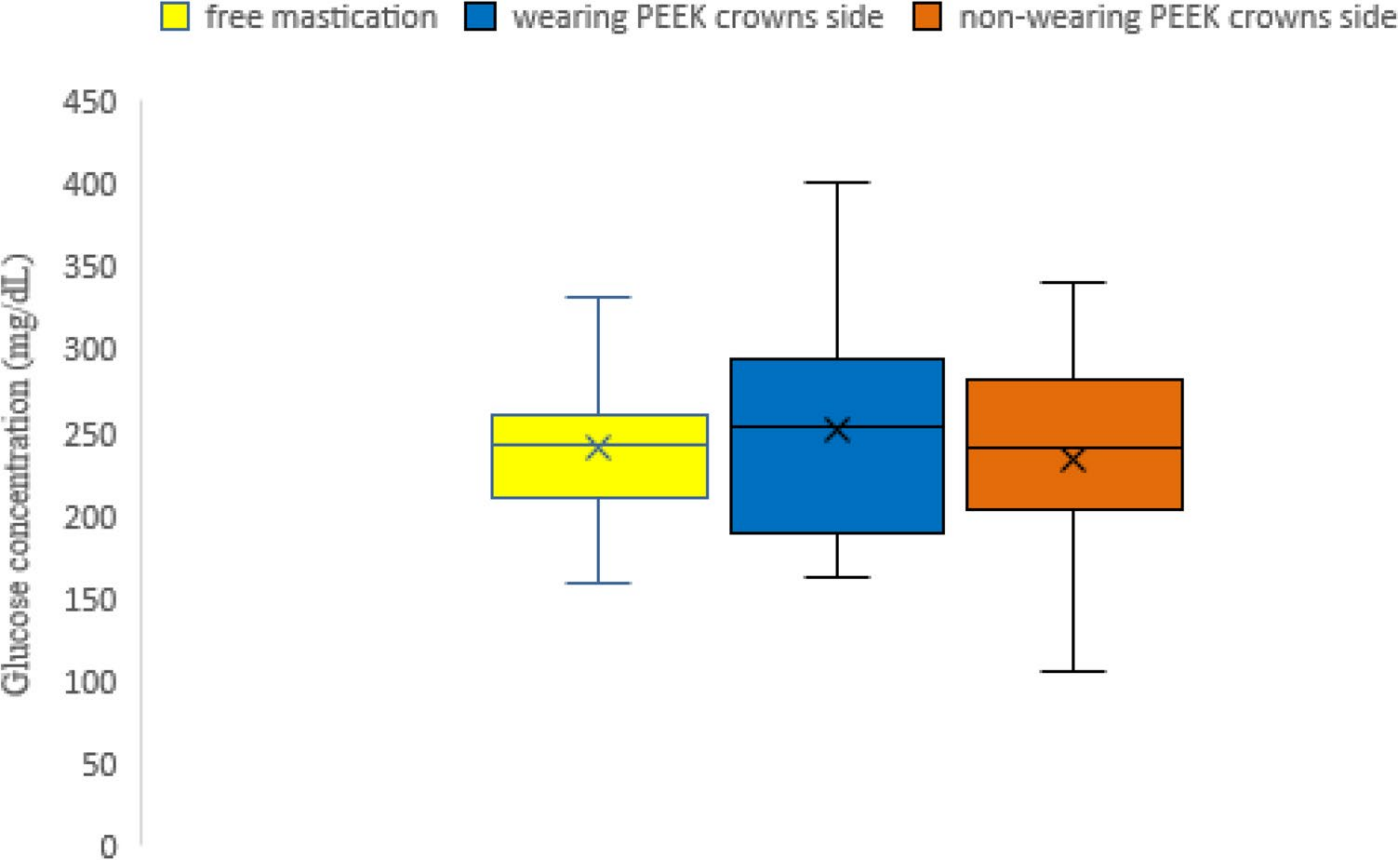
Boxplot of Glucose concentration by masticatory ability test in wearing PEEK side and non-wearing PEEK side. Box; inter quartile range (IQR), Lower whisker; minimum, upper whisker; maximum, Center line; median, cross mark; mean. *Mann–Whitney *U* test shows no significant difference between PEEK and non-PEEK wearing sides.

**Figure 7:**
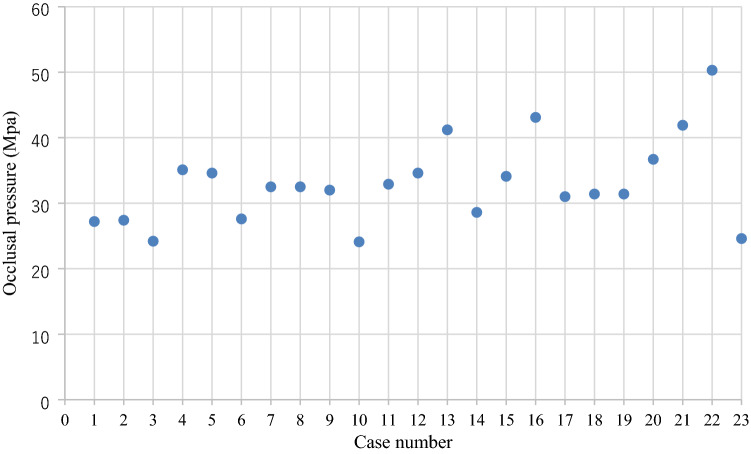
Distribution for the 23 Cases of Occlusal pressure evaluated by Dental prescale II system. *Plots represent mean Occlusal pressure for each case.

**Figure 8:**
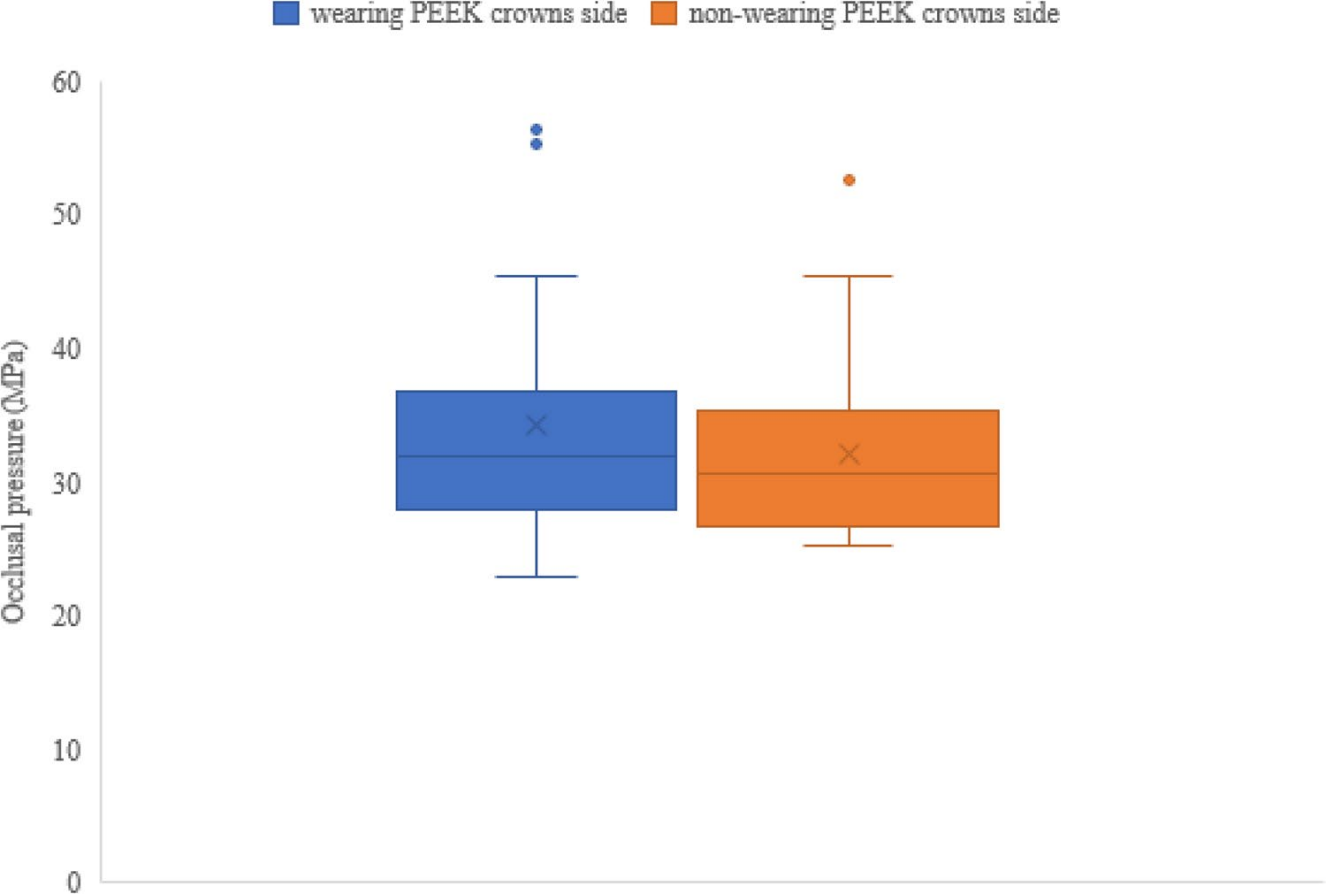
Boxplot of Occlusal pressure in wearing PEEK crowns side and non-wearing PEEK crowns side. Box; inter quartile range (IQR), Lower whisker ; minimum, upper whisker ; maximum, Center line; median, cross mark; mean. The blue and orange dots represent outliers. *Mann–Whitney U test shows no significant difference between PEEK and non-PEEK wearing sides.

**Figure 9:**
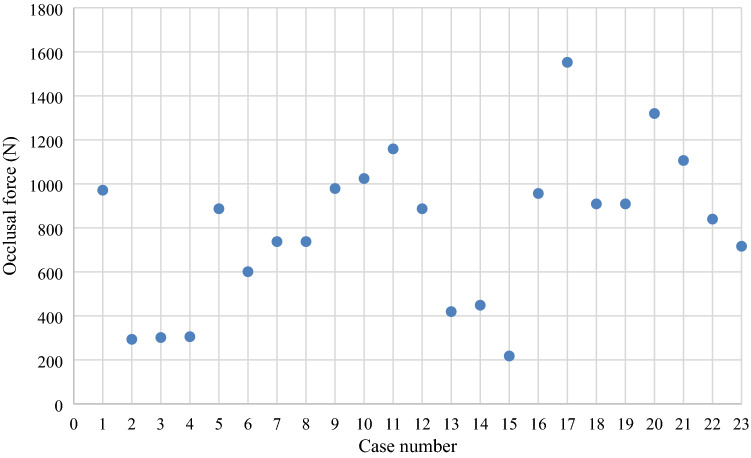
Distribution for the 23 subjects of Occlusal force evaluated by Dental prescale II system. *Plots represent mean Occlusal force for each case.

**Figure 10:**
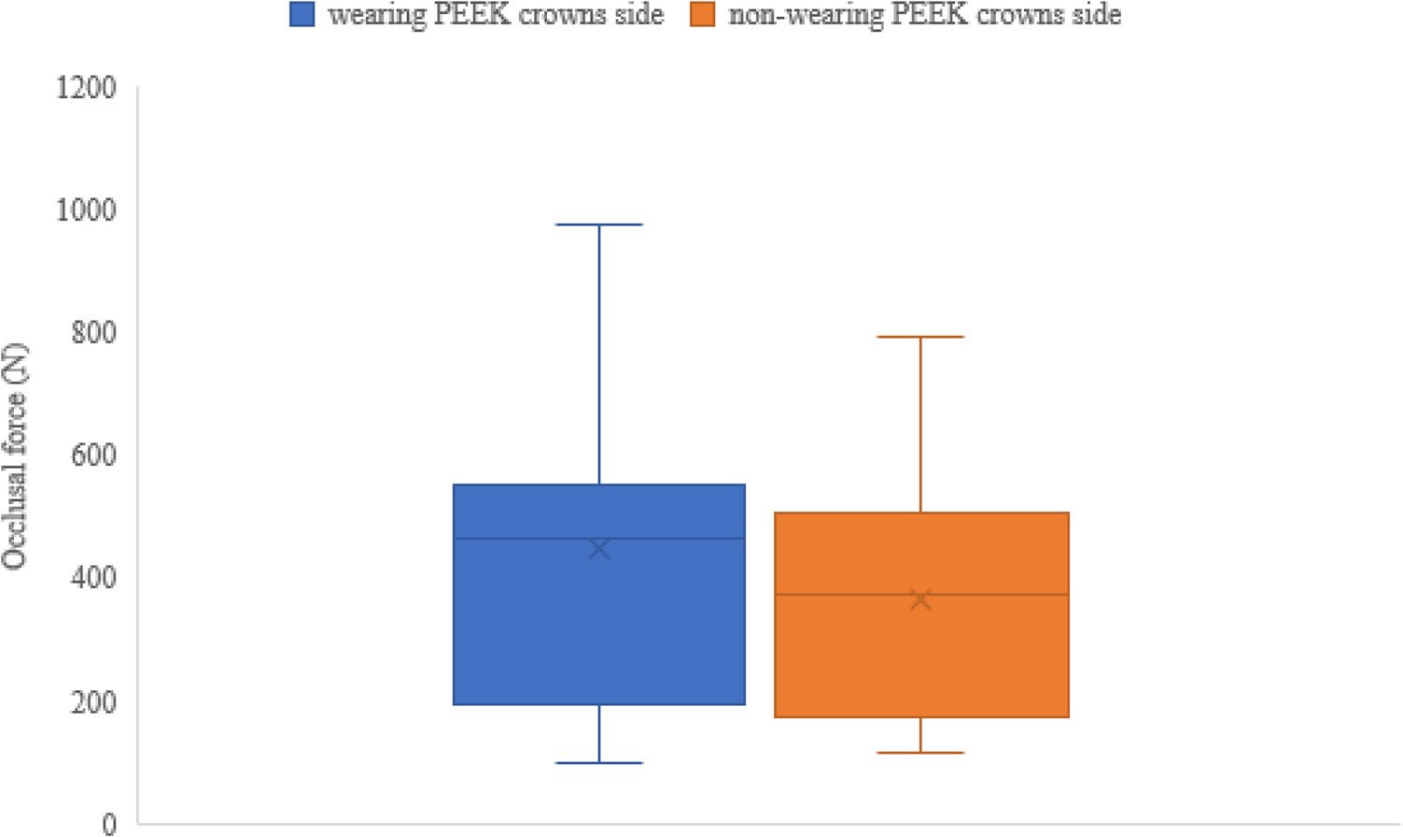
Boxplot of Occlusal force in wearing PEEK crowns side and non-wearing PEEK crowns side. Box; inter quartile range (IQR), Lower whisker; minimum, upper whisker; maximum, Center line; median, cross mark; mean. *Mann–Whitney *U* test shows no significant difference between PEEK and non-PEEK wearing sides.

The results of the masticatory ability test using gummy bears are shown in Fig. 5. The mean values of the 23 cases were 245.2 mg/dL for free mastication, 254.2 mg/dL for wearing PEEK crown side mastication, and 236.1 mg/dL for non-wearing PEEK crown side mastication. There was no statistically significant difference in the masticatory ability test between wearing and non-wearing PEEK crowns sides (*p* = 0.6164) (Fig. 6). The results of the occlusal force measurement are shown in Figs. 7, 8, 9 and 10. The mean pressure and occlusal force of the 23 cases were 33 MPa and 794.5 N, respectively. There was no statistically significant difference in occlusal pressure and occlusal force between wearing and non-wearing PEEK crowns sides (*p* = 0.4734, *p* = 0.2733) (Figs. 8 and 10).

No statistically significant differences were observed in masticatory ability, occlusal presser, and occlusal force due to differences in cement type.

## Discussion

In this study, all subjects were ≥ 20 years of age, had single crown, opposing teeth, and abutment teeth of 3 mm on buccal and lingual sides. In Japan, the age of ≥ 20 years is defined as the age of adulthood in Japan. For the establishment of an occlusal relationship, single crown was used to confirm the wearing condition of a single tooth, the presence of opposing teeth to restore occlusion and masticatory ability through occlusal contact, and a 3 mm height diameter to prevent fracture. The exclusion criteria were planned abutment teeth for partial dentures and teeth with severe periodontal disease that may have a poor prognosis, considering the occurrence of breakage or dehiscence.

Based on the evaluation after six months of cementation (Table 4), the occlusal contact score B was 2 (male: 1, female: 1), occlusal surface condition score B was 2 (male: 2, female: 0), surface texture score B was 6 (male: 3, female: 3), discoloration/staining score B was 7 (male: 2, female: 5), and marginal gingiva score B was 5 (male: 2, female: 3). Table 5 summarizes details of score B evaluation items including bonding methods and abutment and contralateral tooth type. The changes in PEEK surface were significant when the counterparts were zirconia. Super-bond might have caused gingivitis due to the penetration of resin cement into the gingival sulcus during bonding. We speculate that these evaluations might be influenced by the type of antagonist and cementation, but the results were not clear due to small number of cases. Most of the occlusal contact scores were A and point contact was maintained, indicating that the PEEK crowns used in the mouth for 6 months might have had an adequate wear resistance. The results of surface roughness, staining, and gingivitis were poor due to poor polishing method of the PEEK crowns and hence, there is room for improvement. Flat surfaces can be polished and made glossy conditions these methods but polishing after the occlusal adjustment is sometimes inadequate to avoid the risk of losing ridges. Polishing methods for complex shaped areas such as ridges require further research. However, no other problems related to crack, broken, desorption, pain, caries, plaque, the occlusal surface of pairing teeth, contact, and retreatment, were observed. The chewing gum adherence (male: 1, female: 3) was attributed to personal lifestyle (Table 6). Therefore, before treatment with PEEK crowns, the patient should be asked if they are habitual of using chewing gums, and if they are then this method should be avoided. Conversely, evaluation item of aesthetic in nine cases with Good satisfaction score, the proportion of females (male: 1, female: 8) was high. The color tone was milky white, and the esthetic quality was significantly better than that of metal crowns. However, according to subjects’ satisfaction questionnaire results, although the color tone was white, it was far from the color tone of natural teeth and was likely to be identified as an artificial product. The original color tone of PEEK is gray; however, the PEEK crown used in this study is grayish beige. The inclusion of titanium dioxide in the grayish beige allows for a change in color tone. However, it is not possible to impart transparency to PEEK, and no technique has been established that can help match the color tone of natural teeth, although a product that approach the color tone of natural teeth by building up composite resin on the PEEK surface are in the process of development. The PEEK crowns did not delaminate, fracture, or crack, but there was some occlusion, coloration, and surface roughness. The good processability of PEEK might have caused this, but it could also be related to the softness of the material.

In this clinical study, sandblasting, which is widely used in clinical dentistry, was used as a pretreatment for bonding PEEK crowns, a primer and two types of luting cement material, which improve bond strength^18^. Moreover, sandblasting on the inner surface of PEEK improves bond strength^14,15,21,22^. Visio. Link for the inner surface of the PEEK crown, V-Primer for the abutment tooth of the metal core^25^, Super-bond PZ Primer for the abutment tooth of the resin core^26^, and Scotchbond Universal Adhesive for the abutment tooth of the metal and resin cores^27^ were used as primers. This study used the following materials: MMA-based Super-Bond C&B (Sun Medical, Moriyama) and composite-based RelyX Ultimate Resin Cement (3 M ESPE, Neuss). They are commonly used in adhesive resin types of cement or as the optimal priming process after sandblasting the PEEK inner surface and prima treatment with Visio. Link (Bredent, Chesterfield) was published in the literature^19^. Especially, it was used in this study because several papers have reported that Visio. link increases the shear bond strength between PEEK and adhesive resin cement ^19,21,36^. Moreover, MMA-containing resin cement can form chemical bonds with PEEK without surface functionalization. In addition, the CO and COO functional groups on the PEEK surface may react with the Visio. link. We adopted Visio. link as the bonding agent as PEEK surface oxidized by chemical conditioning opens the aromatic rings increases the polarity, and adds functional groups that are more reactive, resulting in increased bond strength. Two types of typical adhesive resin cement (composite and MMA) that are mainly used in clinical practice, were used. In six months, no crown dehiscence was observed with either RelyX Ultimate Resin Cement, a composite cement, or Super-Bond C&B cement, an MMA cement. This suggests that the use of adhesive resin may prevent PEEK crowns from delaminating.

In the masticatory ability test, most of the measured values of free mastication, wearing PEEK crown sided mastication, and non-wearing PEEK crown sided in 20 subjects with 23 cases reached the glucose concentration that is within the healthy range for edentulous people, and it was found that the PEEK crowns provided sufficient masticatory strength. Twenty-three cases had glucose concentrations of 150 mg/dL or higher^30^, which is within the healthy range for edentulous subjects. Furthermore, there was no significant difference in masticatory ability between the same subject with and without PEEK crowns, which suggests that PEEK crowns might not reduce masticatory ability. In addition, the average value of occlusal force was 794.5 N, which was higher than the standard value of 500 N for declining occlusal force. The mean pressure of the case was not significantly lower than 500 N. Six cases were below the standard value of 500 N for occlusal force reduction. In addition, there was no significant difference in occlusal pressure and occlusal force between the wearing and non-wearing PEEK crowns, supporting the fact that there was no significant difference in masticatory ability between the wearing and non-wearing PEEK crowns as well. Therefore, it can be predicted that the subject has an occlusal force within the normal range, and PEEK crowns can withstand occlusal forces.

Regardless of the height of the abutment teeth, no dropouts, fractures, or cracks were observed even after 6 months of placement. However, this result may be limited to only this study because of the subjects' differences in occlusal forces. In some cases, slight wear, staining, roughness, or gingivitis were observed on the surface of the PEEK crowns. This suggests that wear and occlusion might have occurred. However, to our knowledge, no prior studies have reported a relationship between PEEK and gingivitis, but PEEK has been reported to prevent bacterial adhesion^37^. Similar studies include retrospective cohort studies of metal-ceramic crowns and CAD/CAM-produced resin composite crowns^38,39^. Clinical complications occurred in 29.3% of the 362 molar CADCAM resin crowns, of which 74.5% were debonding and 4.7% were fractures. Crown debonding of the molars most frequently occurred in the early stages, within the first 6 months^38^. The most common complications for composite crowns were a fracture of the crown and loss of retention for metal-ceramic crowns^39^. In this study, no debonding and fracture were observed within 6 months. Therefore, PEEK crowns were not inferior to the results of these studies, although at 6 months. These results suggest that PEEK crowns are suitable for continuous patient wear.

Our study had a few limitations. The study did not compare PEEK crown to existing zirconia or metal crowns, so it is unclear whether PEEK crown is superior to existing crowns. We only visually checked the plaque adherence without using staining or a spectrophotometer. Furthermore, in this study, a single evaluator was selected for the purpose of minimizing the variability of evaluation among some evaluators and at multiple time points, but this is debatable. On the other hand, we only did a questionnaire asking patients about their satisfaction after six months of treatment and did not determine the effect of treatment before and after prosthetic treatment using the OHIP form. Therefore, our results might not be objective. The establishment of a chairside polishing method is essential for the clinical application of PEEK crowns, but it has not yet been established. Masticatory ability, occlusal pressure, and occlusal force are valid indices for the entire oral cavity, but specific sites with PEEK crowns could not be tested. Future studies should examine the site where the PEEK crown was placed. Furthermore, masticatory ability, occlusal pressure, and occlusal force were analyzed statistically by continuous correction using the Mann–Whitney *U* test, a nonparametric method. However, we could not validate the differences in masticatory ability, occlusal pressure, and occlusal force between the wearing and non-wearing PEEK crowns due to the sample size. Therefore, future long-term studies with large sample size are required to validate our findings.

## Conclusion

PEEK crowns were fabricated as an alternative to metal crowns due to their biological safety and physical properties. The PEEK crowns were placed with adhesive cement after appropriate treatment of the inner surface of the crowns. Based on our findings on limited subjects, PEEK crowns fabricated using the CAD/CAM system did not fall off, fracture, or had any significant adverse effect on the marginal gingiva or reduction in the ability to masticate after six months of placement, indicating that PEEK is a promising alternative of metal crowns for molars.

## Supplementary Information

Supplementary Information.

## Data Availability

The raw data generated during the study are included in this article and its supplementary file.
